# Environmental constraints for improving motor flexibility during obstacle crossing in older adults

**DOI:** 10.1186/s12984-024-01532-5

**Published:** 2024-12-21

**Authors:** Yuki Suda, Takahiro Higuchi

**Affiliations:** 1 Department of Health Promotion Science, Tokyo Metropolitan University, 1-1 Minami-Osawa, Hachioji, Tokyo, 192-0397 Japan; 2 Japan Society for the Promotion of Science, Tokyo, Japan

**Keywords:** Uncontrolled manifold analysis, Walking, Constraints-led approach, Obstacle avoidance

## Abstract

**Background:**

An age-related decline in motor flexibility, which is the ability to synergistically control the degrees of freedom of the body to ensure stable performance of a task, is a factor that contributes to falls. We investigated whether providing environmental constraints to increase the movement repertoire (i.e., the motor solution that works to achieve one’s goal), in combination with aiming at precise control of the performance, would be effective for improving motor flexibility, and whether the effect on the leading limb would extend to the trailing limb.

**Methods:**

Fifteen older adults (75.1 ± 6.2 years and 14 younger adults (34.6 ± 5.0 years) performed under three walking conditions: walking normally and crossing the obstacle (normal), walking and crossing the obstacle with constraints of foot placement after stepping over it (constrained), and walking and crossing the obstacle with constraints as in the constrained condition, in addition to aiming for maintaining a constant clearance height at the moment of obstacle crossing (precision). An uncontrolled manifold analysis was used to quantify motor flexibility as the synergy index. The foot height at the moment of obstacle crossing was used as the performance variable and seven segmental angles were used as the elemental variables. A higher synergy index indicates greater motor flexibility.

**Results:**

For the leading limb, the synergy index was significantly higher under the precision condition than those under the other conditions. This suggests that not only providing environmental constraints but also keeping constant the performance variable is critical to improving motor flexibility. Moreover, the effects of an increase in the synergy index in the leading limb extended to the trailing limb.

**Conclusions:**

Providing environmental constraints to increase the movement repertoire while also aiming for precision in the performance variable was an effective method of improving motor flexibility during obstacle crossing for older adults.

## Introduction

Many older adults fall when walking and stepping over an obstacle of height [1–3]. Falls mainly occur as a result of tripping with insufficient foot elevation [4, 5]. Notably, falls may also occur as a result of destabilization caused by extraordinary foot elevation, which is a so-called conservative strategy [6]. Destabilization could occur with extraordinary foot elevation in older adults due to the increased amount of time standing on one leg [7–9]. An age-related decrease in the ability to adjust their movement in response to environmental constraints (i.e., adaptive locomotor adjustment) seems to be involved in such inappropriate foot elevation [10, 11].

Sufficient motor flexibility ensures flexible control of body segments to achieve an appropriate foot elevation with a sufficient movement repertoire. Motor flexibility is the ability to synergistically control the abundant degrees of freedom (DoFs) of the body (i.e., the elemental variables) to ensure the stable performance of a task (i.e., the performance variable) [12]. For example, the hip, knee, and ankle joints are coordinated to elevate the toe to a certain height. Even when one joint cannot be used due to injury, motor flexibility leads to maintaining toe elevation at the same height by altering the combination of joint angles. In this way, motor flexibility provides flexibility to react to perturbations (including neuromotor noise or movement errors) and altered task demands while ensuring stable task performance [12].

Quantification of motor flexibility using the uncontrolled manifold (UCM) analysis enables researchers to address the age-related decrease in motor flexibility [13–15] as well as to devise a method of improving it. UCM analysis divides the across-trial variance of the elemental variables into two components: one with variance that has no effect on the performance variable (V_UCM_) and another one that negatively affects the performance variable (V_ORT_) [16, 17]. The V_UCM_ reflects the amount of movement repertoires used to maintain the performance variable, and the V_ORT_ reflects the variability of the performance variable itself. Then, the synergy index (ΔV), representing motor flexibility, is calculated from the two variances (V_UCM_ and V_ORT_). The greater value of ΔV means greater motor flexibility, and ΔV increases as the V_UCM_ becomes relatively higher than the V_ORT_.

Previous studies investigated age-related decreases in motor flexibility using UCM analysis in the reaching task [18], maintaining body balance [15, 19], and stepping over an obstacle [14, 20]. Recently, Suda et al. (2024) demonstrated an age-related decrease in motor flexibility to stabilize the toe height during obstacle crossing for the trailing limb [13]. In this study, older and younger adults were asked to walk for 3 m and crossed an 8 cm obstacle without collision. The UCM analysis was used to quantify motor flexibility during obstacle crossing, with toe height as the performance variable and segment angles as the elemental variables. The results showed that older participants exhibited a significantly lower synergy index (ΔV) during the trailing limb crossing, suggesting an age-related decrease in motor flexibility. Therefore, it is necessary to address the methods of improving the age-related decline in motor flexibility to stabilize the foot height for obstacle crossing.

There is a theoretical idea that is helpful to consider for improving motor flexibility: a constraints-led approach (CLA) [21]. The concept of a CLA is based on the theory of ecological psychology. According to the theory, through the interaction of different constraints induced by the task, environment, and a learner herself/himself, a learner will self-organize in attempts to generate effective movement solutions [21, 22]. A CLA intends to promote the exploration of motor solutions (i.e., movement repertoire) to achieve the desired goal through constraints manipulation. For example, Gray (2018) reported that a CLA approach for teaching baseball batters under a virtual environment was effective for promoting the exploration of motor solutions to achieve an increased launch angle to produce a fly ball [23]. A CLA training in Gray involved adding the constraint of a barrier (i.e., a big wall) that must be hit over and adjusting barrier distance and height based on the participant’s performance. Their results showed that participants in the CLA training group had larger launch angle as compared to other groups in which traditional training was used. More importantly for the purpose of the current study, the variability of the bat path angle during practice was significantly greater in the CLA training group, indicating exploration of motor solutions. From these findings, CLA is a candidate for a method of improving the age-related decline in motor flexibility because it could lead to an increase in motor repertoire as a result of exploration, while the desired, stable task performance is taken into consideration.

Notably, solely providing environmental constraints may be insufficient to improve motor flexibility. This is because environmental constraints could increase not only the movement repertoires but also the variability of the movement that needs to be kept constant (i.e., the performance variable). A previous study showed that the foot height during obstacle crossing was altered due to environmental constraints [24]. In the study, younger participants were asked to step over an obstacle while avoiding a second obstacle placed at ground level beneath it. It was found that toe height varied depending on the position of the second obstacle. This suggests that environmental constraints could lead to variability of the task performance itself. Therefore, it would be necessary to consider environmental constraints that could improve movement repertoires while leading performers to aim at precise control of the performance variable.

In the present study, in order to lead participants to aim at precise control of the performance variable, a target that they touched with the toe of their dominant, right limb was provided above the obstacle. We asked participants to touch the bar only with the leading, right limb, but not the trailing, left limb, based on our expectation that the effects of an intervention on the leading limb would be transferred to the trailing limb. Some studies have indicated that the trailing limb was controlled based on information obtained from the leading limb [25, 26]. For example, Miura et al., (2021) examined whether the foot height over an obstacle was influenced by the contralateral obstacle’s height [25]. Younger participants crossed an obstacle of different heights on either side. The results showed that the foot height for the trailing limb was affected by that for the leading limb. This suggests that there is interaction between the leading and trailing limbs. Thus, if a visual cue for maintaining a consistent clearance height was presented only to the leading limb, we predict that the effect obtained for the leading limb will transfer to the trailing limb.

The first purpose of the present study was to investigate whether providing environmental constraints aimed at increasing the movement repertoire of the whole body (i.e., increasing the V_UCM_) while also aiming at precision in performance (i.e., decreasing the V_ORT_) increases motor flexibility to stabilize foot height (i.e., increasing the ΔV) in older adults. The second purpose was to investigate whether the effect on the leading limb extended to the trailing limb. There were two hypotheses in this study. The first hypothesis is that providing opportunities aimed at increasing movement repertoire (V_UCM_) using a CLA while achieving a consistent clearance height would enhance motor flexibility (i.e., the greater value of the ΔV). The second hypothesis is that an increase in motor flexibility (i.e., the greater value of the ΔV) would extend not only to the leading limb but also to the trailing limb.

## Methods

### Participants

An a priori power analysis was performed based on an analysis of variance using G*power (effect size: f = 0.25, α = 0.05, 1-β = 0.8, number of groups = 2, number of measurements = 3). A sample size of more than 28 participants would be necessary to validate the study’s conclusion. Based on the power analysis, we recruited 15 older adults (seven males and eight females, 75.1 ± 6.2 years) and 14 younger adults (five males and nine females, 34.6 ± 5.0 years). Before performing the main section, participants’ details were collected. The height, leg length (the distance from the greater trochanter to the outer malleolus), and foot length (the distance from toe tip to heel) of each participant were measured in cm, and their weight was measured in kg. The dominant limb, which was defined as the kicking foot, was self-reported [27]. We checked on a self-reported basis that all participants have normal or corrected-to-normal vision, no current musculoskeletal injuries, and no neurological disorders. The cognitive function of older participants was assessed using the Mini-Mental State Examination (MMSE) [28], while their mobility function was assessed using the Timed Up and Go (TUG) test [29]. We confirmed that none of the older adults had any cognitive impairment, using as a cutoff value MMSE > 24 points [28], and any mobility disfunction, using as a cutoff value TUG < 13.5 s [30].

The study was approved by the Ethics Committee of Tokyo Metropolitan University, Japan (H5–22). All methods were carried out in accordance with relevant guidelines and regulations. Written informed consent was obtained from all participants in accordance with the Ethics Committee of Tokyo Metropolitan University and the Declaration of Helsinki.

### Apparatus, Task, and procedures

**Fig. 1:**
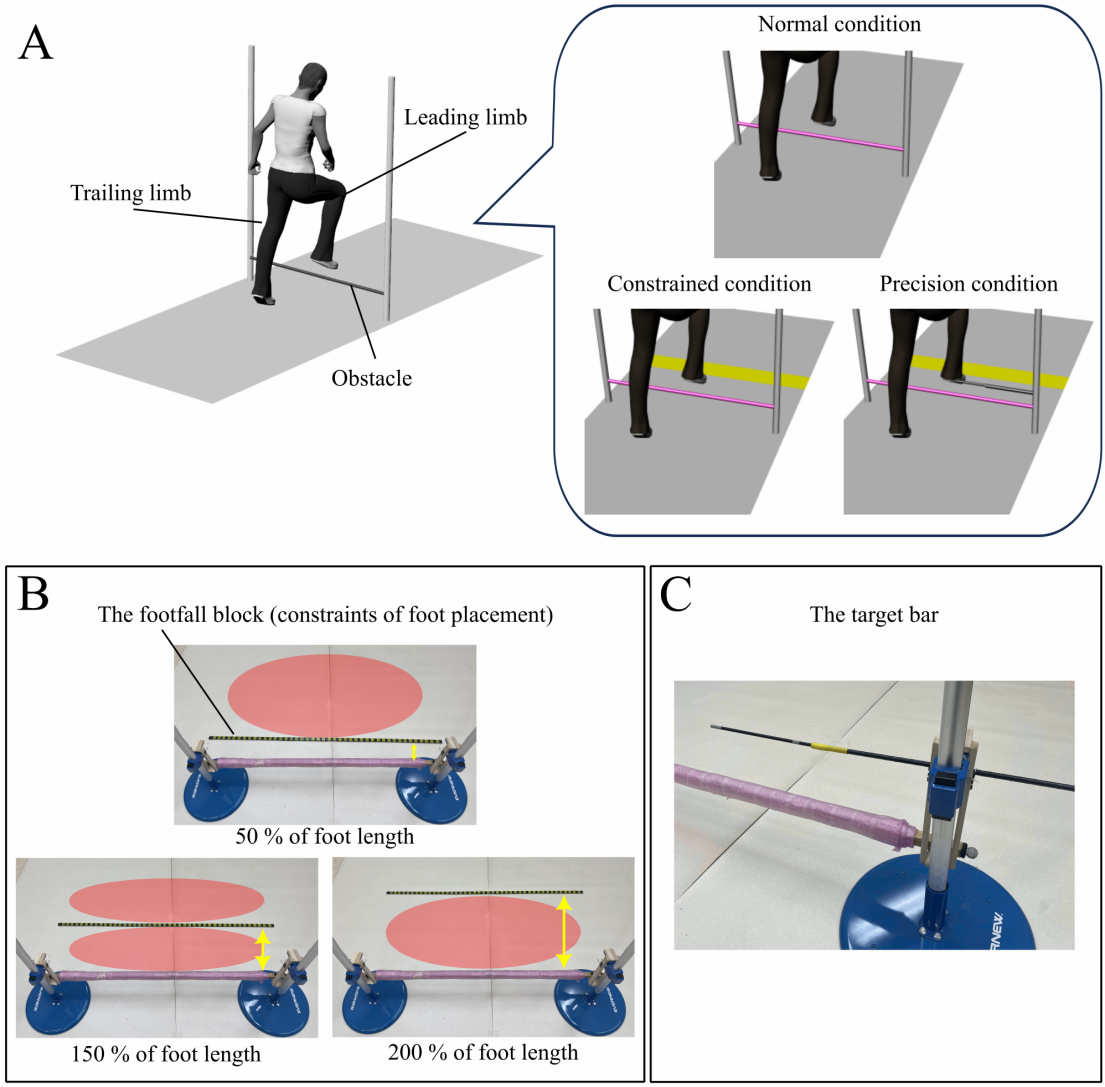
**A**: The procedure of a walking task, performed under three conditions. **B**: The footfall block (shown as a black, dotted line) positioned at an offset 50%, 150%, or 200% of foot length on the landing side of the obstacle for the constrained and precision conditions. Orange ovals show areas where participants can land. **C**: The target bar was set above the obstacle. Participants were asked to touch the target bar with the toe of their dominant, right limb

The present study was conducted in a 6.6 m × 5.6 m room at Tokyo Metropolitan University. The obstacle consisted of two aluminum poles (1.91 m tall and 0.03 m in diameter) and a wooden horizontal bar (1.2 m wide and 0.05 m in diameter) covered with a buffer. The obstacle height was set at 20% of the participant’s leg length (Fig. 1A). A footfall block, which consisted of a wooden bar (0.02 m height, 0.03 m wide, and 1.5 m long), was introduced to constrain foot placement after stepping over the obstacle (Fig. 1B). A target bar, which consisted of wooden bars (0.05 m in diameter and 0.4 m long) and plastic straw (0.06 m in diameter and 0.2 m long), was attached to one of the aluminum poles on the right side, as seen by the participants (Fig. 1C). The target bar was set above the obstacle (a height of 20% of the leg length from the obstacle). The purpose of introducing this target bar was to let participants aim at touching the bar with the toe of their leading, right limb so that the clearance height became constant.

Participants performed a walking task under three conditions (normal, constrained, and precision). All participants initially performed the walking task under the normal condition to avoid any potential consequence from the constraints on foot placement introduced under the other conditions. The order of the constrained and precision conditions was counterbalanced among participants. A total of 45 main trials (15 trails for each condition) were performed. In an effort to perform the UCM analysis in a reliable manner, if participants failed to avoid contact with the obstacle or the footfall block (regarded as an unsuccessful trial), an additional trial was performed to ensure 15 successful trials under each condition. Participants rested for approximately five minutes after performing each condition to avoid the effects of fatigue.

In all conditions, they were asked to cross the obstacle initially with their dominant limb. Since all participants were right-side dominant, the right limb was always the leading limb, and the left limb was the trailing limb. Under the normal condition, participants walked and crossed the obstacle at their own comfortable pace 15 times. Participants performed five practice trials before performing the main trials.

Under the constrained condition, participants walked and crossed the obstacle with constraints on foot placement after stepping over the obstacle. A footfall block was located in one of the three predetermined locations: 50%, 150%, and 200% of the foot length offset on the landing side of the obstacle (Fig. 1B). Participants were asked to adjust their locomotion patterns to avoid stepping onto the footfall block. When the footfall block was located at 50% of the foot length offset, participants needed to extend the length of their step. When the footfall block was located at 150% of the foot length offset, participants needed to choose between lengthening or shortening the length of their step. When the footfall block was located at 200% of the foot length offset, participants needed to shorten the length of their step. For each trial, the location of the footfall block was randomly selected (each location was selected for five trials). Participants were instructed to walk and cross the obstacle comfortably while avoiding contact with the obstacle of height and the footfall block located on the ground by either lengthening or shortening their step. Participants performed five trials with the footfall block in each of the three places. Before performing the main trials, participants completed five practice trials (one trial for 150% of the foot length offset and two trials for each of 50% and 200% of the foot length offset) to familiarize themselves with the task.

Under the precision condition, the procedure was the same as that under the constrained condition, except that a target bar was set above the obstacle to step over. Participants were instructed to walk and cross the obstacle comfortably, avoid contact with the obstacle and the footfall block, and aim to touch the target bar with the toe of their leading limb to maintain a consistent clearance height among all trials.

### Data collection

The kinematic data related to the behavior of stepping over an obstacle was recorded with a 17-camera Qualisys motion analysis system (OQUS 300, Qualisys, Sweden), at a sampling rate of 120 Hz. The kinematic data was low-pass filtered at 6 Hz with a fourth-order Butterworth algorithm. Fourteen reflective markers were placed on both sides of the body as follows: the anterior superior iliac spine, the posterior superior iliac spine, the greater trochanter, the lateral femoral condyles, the lateral malleolus, the calcaneus, and the second metatarsal. Two additional reflective markers were also placed on the edge of the obstacle. All data analysis was conducted using MATLAB (R2023a, MathWorks Inc., Natick, MA, USA). We defined the moment of obstacle crossing as the moment when the marker on the toe crossed the marker on the obstacle in the anterior–posterior (AP) direction. Foot clearance was calculated as the vertical (V) distance between the markers on the obstacle and on the toe at the moment of obstacle crossing, and it was normalized to leg length to account for differences between subjects.

### UCM analysis

**Fig. 2:**
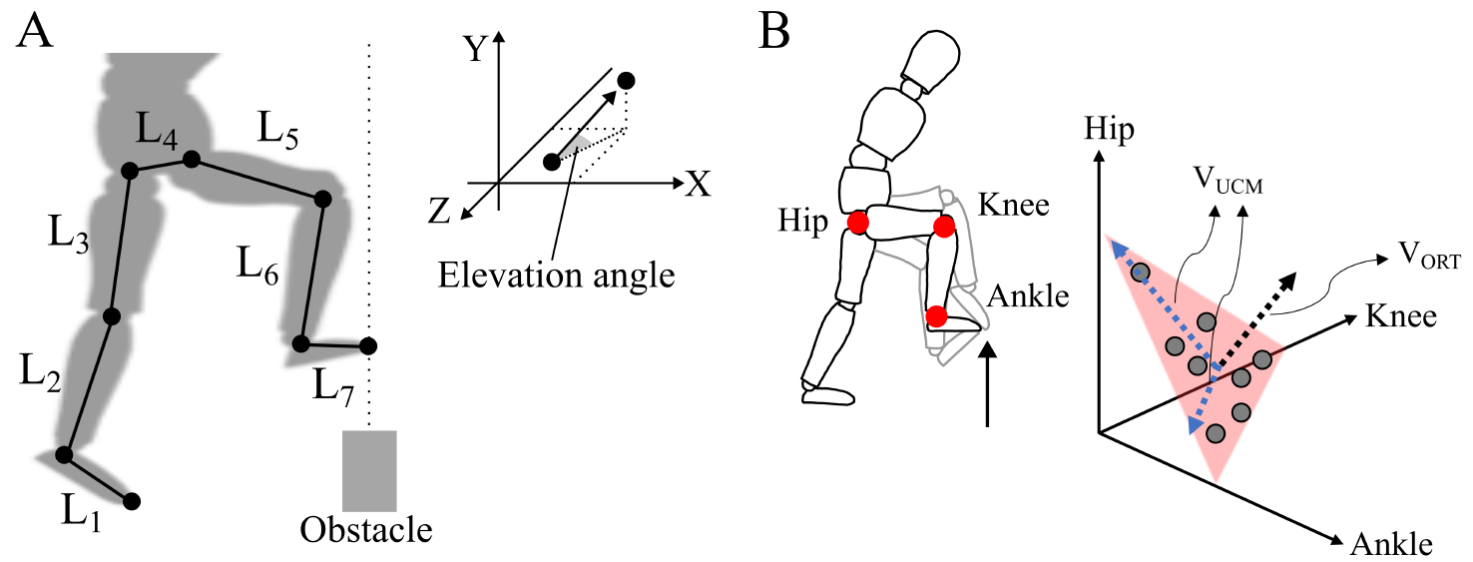
**A**: The geometric model for UCM analysis. L is the length of each segment. Seven segments were defined: each foot (L_1_, L_7_), each shank (L_2_, L_6_), each thigh (L_3_, L_5_), and the pelvis (L_4_). The dotted line shows the moment of obstacle crossing in the leading limb. **B**: Illustration of a three-dimensional example model for UCM analysis. In this model, toe height is the performance variable and hip, knee and ankle joint angles are the elemental variables. If toe height always achieves a target height, the points of combinations of joint angles are plotted in a certain plane. This plane is the UCM space for toe height stabilization, and the variance in parallel with this plane (two directions) is V_UCM_. On the other hand, if toe height does not achieve a constant, the points of combinations of joint angles are not plotted in the plane. The variance in orthogonal of this plane (one dimension) is V_ORT_

We applied UCM analysis to quantify motor flexibility [12, 13, 16]. The protocol of the analysis was based on that of the previous study [13]. The vertical toe position at the moment of obstacle crossing was used as the performance variable. We defined seven segments (right/left foot, right/left shank, right/left thigh, and pelvis) and calculated the elevation angles of each segment as the elemental variables. The kinematic model has seven elevation angles for the V toe position, as follows:

$${\rm{TO}}{{\rm{E}}_{\rm{V}}} = {{\rm{L}}_1}{\rm{si}}{{\rm{n}\alpha}_1} + {{\rm{L}}_2}{\rm{si}}{{\rm{n}\alpha}_2} + {{\rm{L}}_3}{\rm{si}}{{\rm{n}\alpha}_3} + \ldots + {{\rm{L}}_7}{\rm{si}}{{\rm{n}\alpha}_7},$$

where L is the length of each segment, and α is the elevation angle. We show the geometrical model for UCM analysis in Fig. 2A. Then, a Jacobian matrix (J) was calculated as the matrix of partial derivatives relating changes in the elemental variables to changes in the performance variable. The J was evaluated at the mean values of the elemental variables across trials. The null space (E) of the evaluated J provides the basis vectors spanning the J and represents the UCM space. Deviation vectors were calculated as the difference between the elemental variables and their respective means. They were projected onto the UCM space (θ_UCM_) and a space orthogonal to the UCM space (θ_ORT_) using the E vector. The V_UCM_ and V_ORT_ were calculated as the variance of the θ_UCM_ and θ_ORT_ and normalized by DoFs within the UCM and ORT space, respectively. The V_UCM_ represents the movement repertoire and the V_ORT_ represents the task performance itself. To help understand the UCM analysis, a three-dimensional example model is shown in Fig. 2B (it is noted that the seven-dimensional model was actually used in the current study). As the index of motor flexibility, the synergy index (ΔV) was calculated as (V_UCM_ - V_ORT_) / (V_UCM_ + V_ORT_), and it was transformed using Fisher’s z-transformation (ΔVz) [31]. The greater value of ΔVz means greater motor flexibility.

### Statistical analysis

Prior to performing statistical analyses, the normality of the continuous variable had been assessed using the normal Q–Q plot. The results showed that the V_UCM_ and V_ORT_ were not normally distributed. Therefore, we used log-transformation [32]. To identify the characteristics of the participants, a Mann–Whitney U test was used to compare participants’ characteristics (height, weight, and leg length), excluding gender. A Pearson’s chi-squared test was conducted to compare the gender ratio. To test the hypothesis that the precision condition would result in increased motor flexibility, we ran a two-way (group and condition) analysis of variance (ANOVA) with a repeated measure of condition (normal, constrained, and precision). The mean normalized foot clearance, Log(V_UCM_) (the movement repertoire), and Log(V_ORT_) (the variability of the performance variable itself) were also statistically tested using a two-way ANOVA. When the main effect or interaction was significant, we performed post hoc comparisons using the Bonferroni method. The significance threshold was set at 0.05. We also reported the effect size, η_p_^2^. Software package SPSS (version 29.0) was used to conduct all statistical analyses.

## Results

Participants’ characteristics are summarized in Table 1. There was no significant difference between older and younger adults in any characteristic (Table 1). Although not significant, the leg length was marginally different between the two groups (*p* = .077). All participants were right-limb dominant.

**Table 1:** *Participants’ details: mean ± standard deviation*

	Older adults (*n* = 15)	Younger adults (*n* = 14)	*p*-value
Gender (male/female)^a^	7/8	5/9	*p* = .710
Age (years)	75.13 ± 6.23	34.64 ± 5.00	*-*
Dominant limb (right/left)	15/0	14/0	*-*
Height (cm)^b^	160.37 ± 8.05	164.91 ± 9.56	*p* = .425
Weight (kg)^b^	58.34 ± 9.09	58.52 ± 11.62	*p* = .847
Leg length (cm)^b^	71.67 ± 4.47	75.00 ± 3.82	*p* = .077
TUG (sec)	7.52 ± 1.35	*-*	*-*
MMSE (points)	29.53 ± 0.64	*-*	*-*

TUG = Timed Up and Go test; MMSE = Mini-Mental State Examination

a: Pearson’s chi-square test, b: Mann–Whitney U test

In a total of 1305 trials, there were two unsuccessful trials in which a collision occurred under the precision condition, one by an older participant and one by a younger participant. Five older participants had a trial in which they failed to avoid stepping onto the footfall block under the precision condition.

**Fig. 3:**
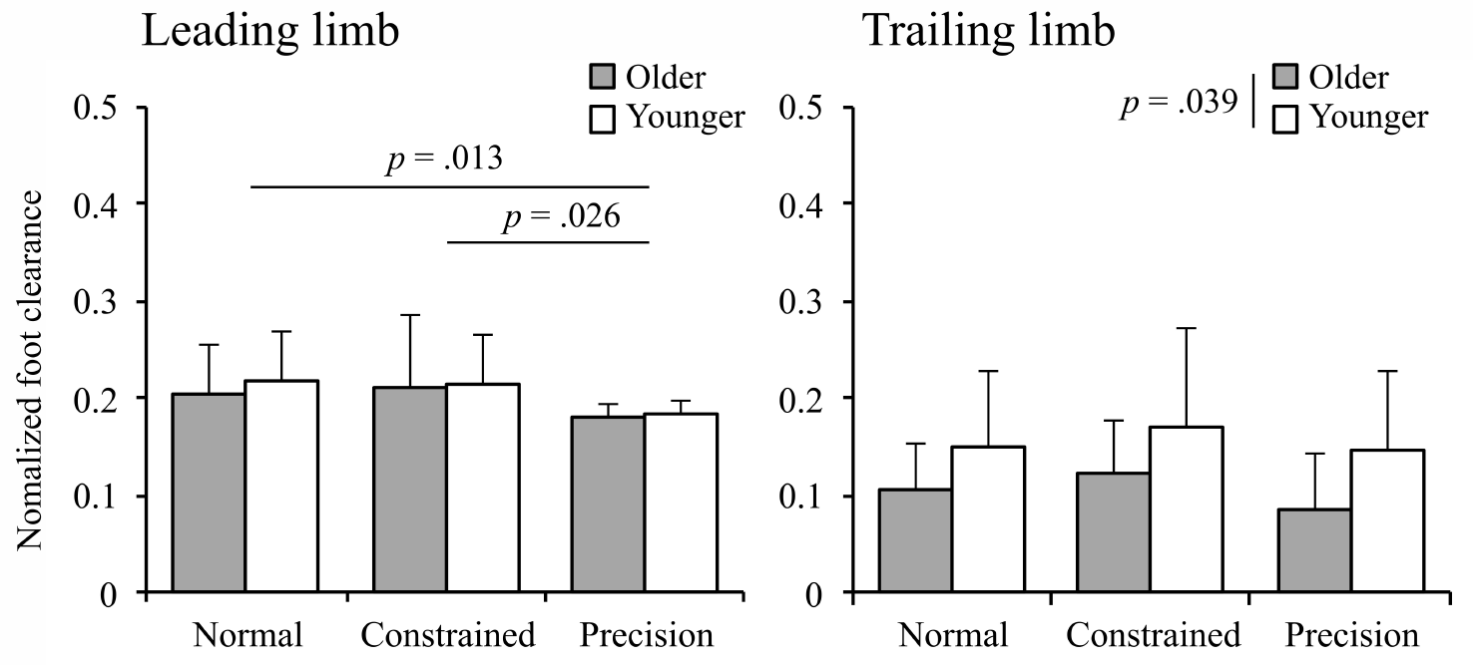
Graphs of the normalized foot clearance in the leading and trailing limbs. Error bars represent the standard deviation

Figure 3 shows the mean normalized foot clearance for the leading and trailing limbs. For the leading limb, an ANOVA showed the main effect of condition was significant (*F*(2,54) = 7.05, *p* = .002, η_p_^2^ = 0.21). Post hoc comparisons showed that the mean normalized foot clearance under the precision condition (0.182 ± 0.003; mean ± standard error) was significantly lower than that under the constrained (0.214 ± 0.012) and normal (0.212 ± 0.010) conditions. No significant main effects of age (*F*(1,27) = 0.21, *p* = .654, η_p_^2^ = 0.01) and interaction (*F*(2,54) = 0.20, *p* = .820, η_p_^2^ = 0.01) were found. For the trailing limb, an ANOVA showed the main effect of condition was significant (*F*(2,54) = 3.42, *p* = .040, η_p_^2^ = 0.11). However, post hoc comparisons showed no significant difference among the conditions. An ANOVA for the trailing limb also showed the main effect of age was significant (*F*(1,27)= 4.72, *p* = .039, η_p_^2^ = 0.15). For the trailing limb, post hoc comparisons showed that the mean normalized foot clearance of younger adults (0.105 ± 0.016) was higher than that of older adults (0.157 ± 0.017). No significant interaction (*F*(2,54) = 0.40, *p* = .670, η_p_^2^ = 0.02) was found.

**Fig. 4:**
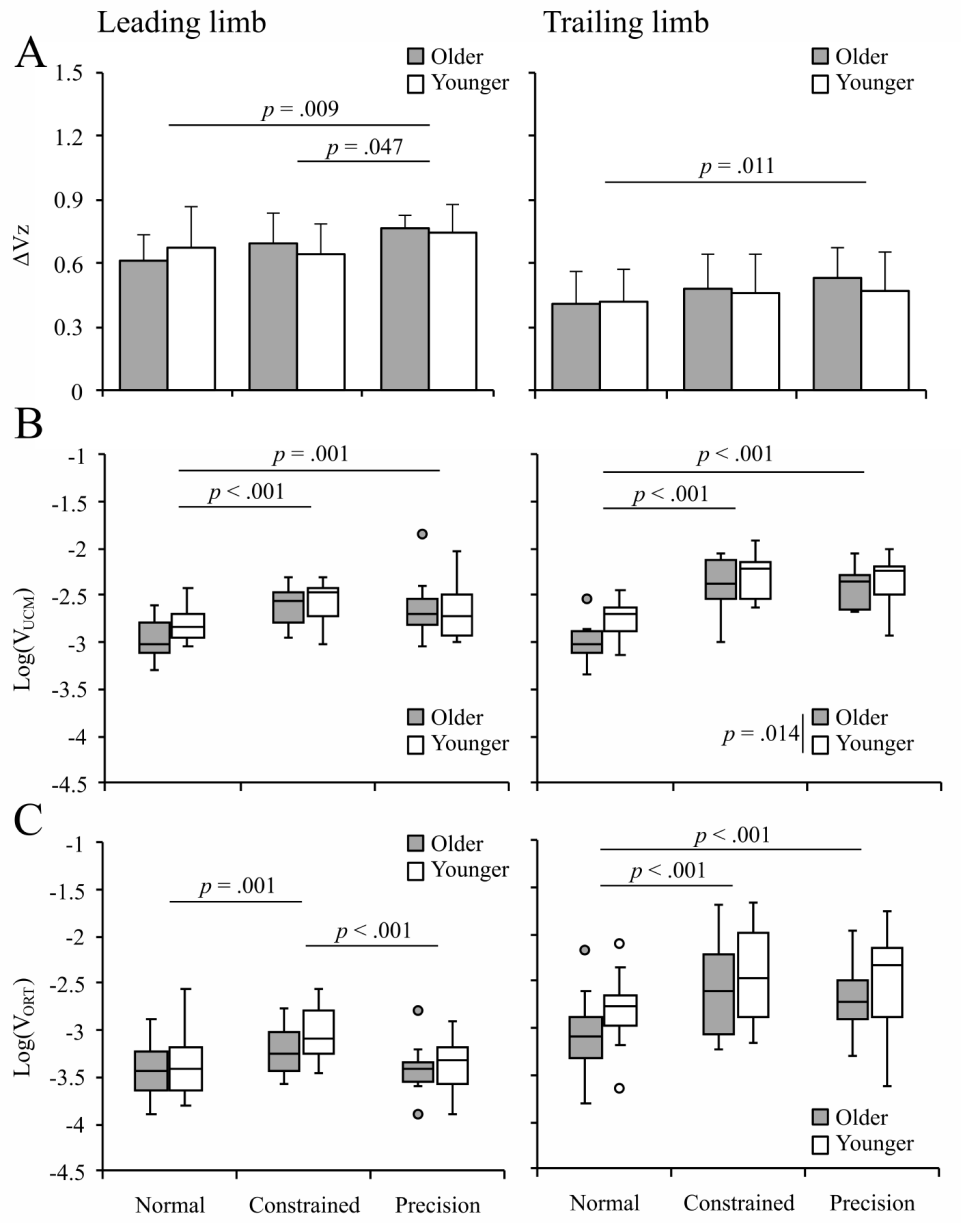
**A**: Graphs of the ΔVz in the leading (left panel) and trailing limbs (right panel). **B**: Graphs of the Log(V_UCM_) in the leading (left panel) and trailing limbs (right panel). **C**: Graphs of the Log(V_ORT_) in the leading (left panel) and trailing limbs (right panel). In all graphs, error bars represent the standard deviation

The mean ΔVz (motor flexibility) is shown in Fig. 4A. For the leading limb, the main effect of condition was significant (*F*(2,54) = 5.97, *p* = .005, η_p_^2^ = 0.18). Post hoc comparisons showed that the ΔVz under the precision condition (0.754 ± 0.019) was significantly higher than that under the constrained (0.667 ± 0.027) and normal (0.642 ± 0.030) conditions. This indicates that the ΔVz increased 13% under the precision condition relative to the constrained condition and 17% relative to the normal condition. No significant main effects of age (*F*(1,27) = 0.004, *p* = .953, η_p_^2^ = 0.00) and interaction (*F*(2,54) = 1.69, *p* = .194, η_p_^2^ = 0.06) were found. For the trailing limb, an ANOVA of the mean ΔVz showed the main effect of condition (*F*(2,54) = 4.23, *p* = .020, η_p_^2^ = 0.14). Post hoc comparisons showed that the ΔVz under the precision condition (0.502 ± 0.031) was significantly higher than that under the normal condition (0.417 ± 0.029). This indicates that the ΔVz increased 20% under the precision condition relative to the normal condition. No significant main effects of age (*F*(1,27) = 0.26, *p* = .618, η_p_^2^ = 0.01) and interaction (*F*(2,54) = 0.58, *p* = .564, η_p_^2^ = 0.02) were found. Collectively these results indicate that, regardless of age, motor flexibility under the precision condition increased as compared to that under the normal and constrained conditions.

The mean V_UCM_ (movement repertoire) is shown in Fig. 4B. Graphs show the transformed data (Log(V_UCM_)) because the V_UCM_ was not normal. For the leading limb, the main effect of condition was significant (*F*(2,54) = 16.6, *p* < .001, η_p_^2^ = 0.38). Post hoc comparisons showed that the mean Log(V_UCM_) under the normal condition (-2.894 ± 0.035) was significantly lower than that under the precision (-2.658 ± 0.053) and constrained (-2.571 ± 0.040) conditions. No significant main effects of age (*F*(1,27) = 1.57, *p* = .221, η_p_^2^ = 0.06) and interaction (*F*(2,54) = 1.30, *p* = .282, η_p_^2^ = 0.05) were found. For the trailing limb, the main effect of condition was significant (*F*(2,54) = 66.7, *p* < .001, η_p_^2^ = 0.71). Post hoc comparisons showed that the mean Log(V_UCM_) under the normal condition (-2.882 ± 0.035) was significantly lower than that under the precision (-2.383 ± 0.042) and constrained (-2.339 ± 0.047) conditions. The main effect of age was also significant (*F*(1,27) = 6.83, *p* = .014, η_p_^2^ = 0.20). The Log(V_UCM_) in younger adults (-2.460 ± 0.041) was higher than that in older adults (-2.609 ± 0.040). No significant interaction (*F*(2,54) = 1.79, *p* = .177, η_p_^2^ = 0.06) was found. Collectively these results indicate that, regardless of age, the movement repertoire under the precision and constrained conditions increased as compared to that under the normal condition.

The mean V_ORT_ (the variability of the performance variable itself) is shown in Fig. 4C. As in the V_UCM_, graphs show the transformed data (Log(V_ORT_)). For the leading limb, the main effect of condition was significant (*F*(2,54) = 11.1, *p* < .001, η_p_^2^ = 0.29). Post hoc comparisons showed that the mean Log(V_ORT_) under the precision condition (-3.387 ± 0.050) was significantly lower than that under the constrained condition (-3.128 ± 0.049), and the mean Log(V_ORT_) under the normal condition (-3.399 ± 0.056) was significantly lower than that under the constrained condition. No significant main effects of age (*F*(1,27) = 1.08, *p* = .309, η_p_^2^ = 0.04) and interaction (*F*(2,54) = 0.57, *p* = .567, η_p_^2^ = 0.02) were found. For the trailing limb, the main effect of condition was significant (*F*(2,54) = 12.3, *p* < .001, η_p_^2^ = 0.31). Post hoc comparisons showed that the mean Log(V_ORT_) under the normal condition (-2.935 ± 0.070) was significantly lower than that under the precision (-2.611 ± 0.083) and constrained (-2.511 ± 0.089) conditions. No significant main effects of age (*F*(1,27) = 2.66, *p* = .115, η_p_^2^ = 0.09) and interaction (*F*(2,54) = 0.17, *p* = .844, η_p_^2^ = 0.01) were found. Collectively these results indicate that, regardless of age, the variability of task performance under the precision condition was not increased as compared to the normal condition.

## Discussion

The purpose of the present study was twofold: first, we addressed whether providing environmental constraints aimed at increasing the movement repertoire of the whole body (i.e., increasing the V_UCM_) while also aiming at precision in performance (i.e., decreasing the V_ORT_) would increase motor flexibility to stabilize foot height (i.e., increasing the ΔVz) in older adults. Second, we also addressed whether the effect on the leading limb would be extended to the trailing limb. The present findings indicate that motor flexibility to stabilize the toe height at the moment of obstacle crossing increased under the precision condition (Fig. 4A, left panel). This supported the first hypothesis: providing environmental constraints to increase the movement repertoire for achieving a consistent clearance height enhanced motor flexibility. Moreover, the effects of an increase in motor flexibility in the leading limb was also observed for the trailing limb (Fig. 4A, right panel), which supported the second hypothesis. These findings suggest that providing environmental constraints aimed at increasing the movement repertoire of the whole body while also aiming at precision in performance increases motor flexibility in older adults.

Providing environmental constraints to aim at increasing the movement repertoire while aiming for precision in the performance variable (i.e., the precision condition) was an effective method for increasing motor flexibility during obstacle crossing in both older and younger adults. In the leading limb, increased synergy index (ΔVz) was accompanied by an increase in the V_UCM_ (as we could see with the increase of the V_UCM_ under the precision condition in Fig. 4B, left panel) and a suppression of the rise in the V_ORT_ (as we could see as the V_ORT_ remains the same under the precision condition in Fig. 4C, left panel). Wu and Latash (2014) showed three scenarios for increased motor flexibility: (a) V_UCM_ increases but V_ORT_ decreases or remains the same, (b) V_UCM_ remains the same but V_ORT_ decreases, (c) V_UCM_ decreases less than V_ORT_. Scenario (a) is considered to be ideal because increasing V_UCM_ (i.e., the movement repertoire) is useful for flexibly controlling the body [33]. The method of the precision condition in the present study was ideal in that an increase in the V_UCM_ and a suppression of the V_ORT_ increase during obstacle crossing were encouraged simultaneously. The present findings also showed that solely providing environmental constraints (constrained condition) did not lead to an increase in motor flexibility, with the result that ΔVz under the constrained condition did not increase significantly as compared to that under the normal condition in Fig. 4A. This was consistent with the findings of previous studies [34, 35]. In a recent study on upper limb reaching, younger participants were asked to manually point to a target while moving over an obstacle for pre- and post-tests [34]. In practice, the participants performed target-pointing movements while moving over an obstacle of different heights to adjust the trajectory of their upper limb (i.e., environmental constraints). The findings indicated no improvement in motor flexibility post-practice [34]. In another study in which individuals with cerebellar ataxia were asked to walk, the lower limb could be pulled suddenly as a perturbation [35]. Again, the results showed no significant improvements in motor flexibility after intervention [35]. These findings suggest that environmental constraints alone were an insufficient method of improving motor flexibility. Based on the present findings, we believe that two factors are necessary for improving motor flexibility: increasing the movement repertoire and keeping the performance variable to be constant.

We found that the movement repertoire increased in conditions that included environmental constraints (constrained and precision conditions), as illustrated in Fig. 4B, with the increase of the V_UCM_. This suggests that environmental constraints contribute to increasing the movement repertoire. Moreover, our findings were in line with those of previous studies using the CLA approach [21–23]. The CLA approach promotes the exploration of motor solutions to achieve the goal of the task. We believe that the movement repertoire would be increased as a result of exploring movement to achieve the task through constraints manipulation. Environmental constraints were an effective method of increasing the movement repertoire (i.e., V_UCM_).

Interestingly, although the environmental constraints were imposed after obstacle crossing, there was an increase in the movement repertoire even before the moment of crossing. Stepping over an obstacle involves movement adjustments prior to the moment of obstacle crossing, such as decreased walking speed or adjusted step length [10, 36]. Other studies reported that, when there are constraints on lower limb placement after obstacle crossing, movement adjustments occur not only after clearing an obstacle but also at the moment of obstacle avoidance [24, 37]. These suggest that environmental constraints on the landing position effectively increased the movement repertoire at the moment of obstacle crossing.

For the leading limb, the results showed that the V_ORT_ was significantly lower under the precision condition as compared to the constrained condition, as we can see in Fig. 4C, left panel. This suggests that touching the target bar at the moment of obstacle crossing ensured the accuracy of the performance variable. In studies involving force production or reaching tasks, clear goals—such as target force or target position—help ensure the accuracy of the performance variable [34, 38–40]. Since motor flexibility involves achieving the same single action through various movement repertoires, providing a target to ensure accuracy in obstacle crossing is likely to help improve motor flexibility during stepping over an obstacle.

Solely aiming for precision in the performance variable does not necessarily contribute to increased motor flexibility because the performance can be precise even with simple patterns of movements (i.e., single set of joint coordination to perform a task). Suda et al. (2024) reported the possibility that the repetition of the simple movement pattern would eventually lead to a decline in motor flexibility (i.e., the decrease of ΔV) [13]. To increase motor flexibility, exploring various movement options to achieve the same performance would be a key issue. A previous study [39] investigated the intervention effect on motor flexibility for reaching. In their study, a perturbation was provided during practice, compelling participants to modify their movements to reach straight toward a target. The results showed that the V_UCM_ increased while the V_ORT_ remained constant, increasing motor flexibility. This suggests that altering the combination of joint angles in response to perturbation to achieve the same, straight trajectory to a target leads to the increased movement flexibility. Generally, when a motor solution is achieved with a certain pattern of movement, a learner will reproduce this movement to become experienced. Once experienced, the learner will try to apply it in different environmental contexts. This leads the learner to explore various movement patterns to adapt to the different contexts, providing a change that increases motor flexibility.

The effects of an increase in motor flexibility in the leading limb extended to the trailing limb, which was not directly manipulated (Fig. 4A). Several studies have indicated that, in stepping over an obstacle, there is an interaction between the leading and trailing limbs in motor control [25, 26]. Hagio and Kouzaki (2020) indicated that limb-specific motor memories for obstacle crossing movements in the leading and trailing limbs were shared, and the central nervous system controls the trailing limb based on the visuomotor information in the leading limb [26]. Regarding the reason why the intervention’s effect on the leading limb resulted in increased motor flexibility in the trailing limb, we believe that the trailing limb was controlled based on the motor memory of the leading limb. Motor flexibility increased in the trailing limb despite the absence of specific manipulation, such as aiming for precision in the performance variable. Therefore, it can be said that participants used information about the movement learned by the leading limb.

Although the V_UCM_ in the trailing limb showed changes similar to those in the leading limb (Fig. 4B), the V_ORT_ in the trailing limb increased under the precision condition as compared to that in the normal condition (Fig. 4C). Even though the information from the leading limb was utilized in the trailing limb, a lack of visual information may have led to an increase in the variability of the performance variable [41]. Interventions that suppress an increase in the V_ORT_, such as providing real-time feedback on the trailing limb [26, 42], could enhance the accuracy of the trailing limb height and may provide a more effective method of increasing motor flexibility for the trailing limb.

Our results indicated that experience in the precision condition would be effective for increasing motor flexibility, not only in older adults but also in younger adults (Fig. 4A). A possible explanation for similar effects between older and younger participants is that older participants in our present study had relatively high physical/cognitive function. In fact, by the performance of TUG, older participants in our study showed relatively high mobility functions, indicating their lower risk of falls. As compared to older adults with lower physical/cognitive function, older adults with higher physical/cognitive function are characterized by relatively higher motor flexibility as a baseline. As a result, learning gain was not that high, leading to results comparable to those of younger participants. Considering that the mobility function is positively correlated with motor flexibility [12, 19], such an explanations is somewhat reasonable.

Improvement in motor flexibility is expected to have a significant impact on older adults from the aspect of adaptability. Based on the dynamical systems theory, one age-related change is the loss of adaptability [43, 44]. This leads to difficulty in adapting behavior to task demands and is a factor contributing to falls. Previous studies have reported that healthy older adults are unable to change their movements in response to task demands, suggesting a loss of adaptability [44–46]. The cause of the loss of adaptability has been believed to reduce the capacity to adjust the number of DoFs according to task demands, resulting in reliance on simple movement patterns [43]. This suggests that the loss of adaptability would be related to a decline in motor flexibility, at the point of utilizing the DoFs. Thus, improvement in motor flexibility by the precision condition is expected to positively affect adaptability in older adults. These effects may be expected to improve the loss of adaptability associated with aging, in contrast to irreversible changes associated with aging (nerve degeneration and musculoskeletal deterioration).

While this study focused on an immediate increase in motor flexibility, further investigation into retention and transfer effects is necessary. Some previous studies have shown the retention and transfer effects on learning motor flexibility in the reaching task [39, 47], a force-production task [38, 48], a two-finger force-exertion task [40, 49], upright balance [50] and gait [51]. For example, Eckardt and Rosenblatt (2019) investigated whether resistance training in unstable environments could promote motor flexibility during walking [51]. Older participants were randomly assigned to one of three training groups: stable whole-limb machine-based training, unstable free-weight training, and stable machine-based adductor/abductor training. After the training of each group, motor flexibility during gait was improved in the unstable free-weight training group [51]. Yang et al. 2007 reported that the retention and transfer effects on motor flexibility in reaching movement were found in younger participants [39]. Wu et al. 2013, also reported that in a task in which two fingers exert the same force as the target, a long-term intervention effect was found in both older and younger subjects [40]. Among them, Eckardt and Rosenblatt reported the transfer effects on uneven surface walking after intervention, suggesting real-world transferability. Based on these findings, we believe that motor flexibility is maintained and transferred to real-world scenarios. To establish training methods for improving motor flexibility during obstacle crossing, future research should examine the long-term training effects of the method used in this study.

The characteristics of basic movement during stepping over an obstacle provided some insights. In the leading limb, the results indicated that the normalized mean foot clearance under the precision condition was lower than that under the other conditions (Fig. 3, left panel). We believe that, due to a requirement of 20% of the participant’s leg length for clearance height under the precision condition, participants had to alter their toe height from their natural one. On the other hand, in the trailing limb, while the main effect of condition was not significant, the main effect of age was significant: the mean normalized foot clearance of younger adults was higher than that of older adults (Fig. 3, right panel). This was in line with the findings of previous studies [5, 52]: the foot clearance of younger adults was higher than that of older adults in the trailing limb.

This study had three limitations. First, stabilizing the toe height while crossing an object is not necessary for completing the task; pedestrians are able to avoid collisions with an obstacle without stabilizing the toe height. However, maintaining the foot lift at the appropriate place in trials is a sufficient condition for completing the task. In addition, the foot position at the moment of obstacle crossing has been regarded as a potential control parameter [13, 53, 54]. Therefore, we regarded the position of the toe height as a critical variable and applied the UCM analysis to evaluate movement repertoires. The UCM analysis has been used similarly in relevant previous studies in which precision of performance is not requested, such as during walking [55–57] and during obstacle crossing [53, 58]. Second, since the toe was touching the target bar at the moment of obstacle crossing, the possibility of some physical impact could not be ruled out. We believe that, in an environment without physical stimuli (such as passing through an aperture between two target markers), the task can be challenging. Third, the effect of solely aiming for precision in the performance variable was not examined. The reason for not including this condition was to reduce the number of trials. To apply the UCM analysis, at least 15 trials are needed for each condition. If a condition with just the target bar was added, 60 trials would be required per participant, which would be hard for older adults. However, the design of this study could not conclude whether presenting targets alone is effective. Future studies should examine whether presenting targets alone is effective.

## Conclusion

Our study demonstrated that providing opportunities to increase the movement repertoire while also aiming for precision in the performance variable effectively improved motor flexibility during obstacle crossing in older and younger adults. Solely providing environmental constraints contributes to increasing the movement repertoire but is accompanied by an increase in the variability of the performance variable. Therefore, two factors are necessary to increase motor flexibility: increasing the movement repertoire and keeping the performance variable constant. We believe that evidence from this study introduces a new method for rehabilitation to improve motor flexibility during obstacle crossing, resulting in increased adaptability to environmental properties and a reduced risk of falls in older adults.

## Data Availability

No datasets were generated or analysed during the current study.
